# Proton Pump Inhibitors and Oncologic Treatment Efficacy: A Practical Review of the Literature for Oncologists

**DOI:** 10.3390/curroncol28010076

**Published:** 2021-02-03

**Authors:** Angel A. T. Uchiyama, Pedro A. I. A. Silva, Moisés S. M. Lopes, Cheng T. Yen, Eliza D. Ricardo, Taciana Mutão, Jefferson R. Pimenta, Larissa M. Machado, Denis S. Shimba, Renata D. Peixoto

**Affiliations:** 1 Centro Especializado em Oncologia, Hospital Alemão Oswaldo Cruz, São Paulo 01327-001, Brazil; atuchiyama@haoc.com.br (A.A.T.U.); pismael@haoc.com.br (P.A.I.A.S.); msmlopes@haoc.com.br (M.S.M.L.); ctyen@haoc.com.br (C.T.Y.); edalsasso@haoc.com.br (E.D.R.); tsmutao@haoc.com.br (T.M.); jpimenta@haoc.com.br (J.R.P.); lmachado@haoc.com.br (L.M.M.); dshimba@haoc.com.br (D.S.S.); 2 Centro Paulista de Oncologia, Grupo Oncoclínicas, São Paulo 04538-132, Brazil

**Keywords:** proton pump inhibitors, chemotherapy, immunotherapy, drug-drug interaction, tyrosine kinase inhibitors

## Abstract

Proton pump inhibitors (PPIs) are the most commonly used anti-acid drugs worldwide, including among cancer patients. However, drug-drug interactions between PPIs and other agents may lead to decreased drug absorption with possible reduced therapeutic benefit, or even increased toxicity. Unfortunately, only scarce data exist regarding the safety of concomitant PPI use with anti-cancer agents. We aim at reviewing current evidence on this possible interaction by dividing anti-cancer agents by class. Until further data is available, we encourage healthcare providers to limit unnecessary PPI overuse.

## 1. Introduction

Proton pump inhibitors (PPIs) belong to a family of drugs that bind to the H+ /K+ -ATPases in the gastric oxyntic cells, inhibiting, therefore, acid release [1,2]. They soon became the most widely prescribed drugs for gastroesophageal reflux and peptic ulcer disease worldwide due to their excellent tolerability and safety profile, as well as their higher acid inhibition when compared to older anti-acid drugs such as histamine 2-receptor antagonists (H2RA), anticholinergics, and synthetic prostaglandin analogs [3,4]. However, we have been witnessing a continuous growth of PPI use, which cannot only be explained by the simple substitution for the previous anti-acid drugs. Two factors may explain this alarming increasing utilization of PPIs: an inappropriate prescription by physicians, and the over-the-counter availability of this class of drugs [5,6]. The overuse of PPIs leads to unnecessary costs, long-term adverse events and potential interaction with other drugs.

Scarce literature evidence reports on the prevalence of PPI use among cancer patients. An American retrospective analysis from healthcare databases, comprising more than 1,800,000 individuals, described a prevalence of acid-reducing agents (ARA) use among cancer patients of up to 33% [7], and PPIs were the most commonly prescribed agent among this class of drugs. In fact, the percentage of PPI users is expected to be much higher given its over-the-counter use by millions of patients.

Reduced therapeutic benefit of anti-cancer agents due to decreased drug absorption may be the consequence of drug-drug interactions (DDIs) with PPIs. Increased toxicity may also be encountered. This becomes even more relevant as the availability of oral cancer drugs in the market has been rising exponentially for several reasons: convenience, less stress for patients, less institutional costs and decreased infusion time [8]. However, pharmacokinetic variability of oral anti-cancer agents differs from parenteral drugs. In order to be adequately absorbed, many orally administered drugs rely on pH-dependent solubility to dissolve within the stomach [9]. Gastric pH level elevations induced by PPIs can, for this reason, impair an oral drug’s ability to reach adequate systemic levels. Other mechanisms may also explain DDIs between PPIs and anti-cancer agents, such as altered microbiota induced by PPIs and their metabolism by cytochrome P450 enzymes [10,11].

To date, most data on different interactions between oncologic drugs and PPIs are not cancer-specific. In this review we aim at describing the effects of PPIs in the efficacy of several anti-cancer agents, dividing them by class (Table 1). Although a growing body of evidence suggests both pro- and anti-carcinogenic associations with PPIs, these associations will not be explored in this manuscript.

## 2. Tyrosine Kinase Inhibitors

### 2.1. EGFR TKIs

Gefitinib, erlotinib, afatinib, dacometinib and osimertinib are tyrosine-kinase inhibitors (TKIs) targeting specifically epidermal growth factor receptor (EGFR) [12]. Erlotinib solubility is pH-dependent and concomitant use of this drug with ARA may lead to its reduced absorption [13,14]. Erlotinib PK values decrease upon concomitant administration of ARAs, such as omeprazole and ranitidine. Therefore, concomitant use of these classes of drugs is not recommended in clinical practice. If treatment with an H2RA is required, erlotinib should be administered hours away from ranitidine [12].

Several studies show decreased efficacy of erlotinib when concomitant use of ARAs occurs. The first study to show a deleterious effect was a single-center retrospective analysis of 507 patients with advanced non-small cell lung cancer (NSCLC), in which 124 patients received concomitant ARA. In this unselected population, median (progression-free survival) PFS and (overall survival) OS in ARA vs. non-ARA groups were 1.4 vs. 2.3 months (HR = 1.83, *p* < 0.001) and 12.9 vs. 16.8 months (HR = 1.37, *p* = 0.003), respectively [15]. Another retrospective analysis with 76 patients showed lower median PFS among concurrent ARA users compared with non-users (11 vs. 5.3 months, *p* = 0.029), although there was no difference in OS (28.5 vs. 24.7 months, *p* = 0.38) [16]. A more recent analysis with lung cancer patients reported a 21% increased risk of death among individuals receiving erlotinib with a concomitant PPI [17].

On the other hand, retrospective analysis of the randomized study BR21 reported a lack of significant differences in PFS and OS among ARA users and nonusers. However, it is worth mentioning that the population of this study was not selected for patients with EGFR activating mutations [18]. Interestingly, a small randomized, cross-over, PK study with 28 lung cancer patients reported a clinically significant increase in the bioavailability of erlotinib during esomeprazole treatment with cola intake [19].

Gefitinib, also a selective EGFR inhibitor, has a limited solubility at high pH. Therefore, increase in gastric pH with ARAs may decrease its absorption and bioavailability [14]. The clinical impact of the coadministration of ARAs on gefitinib therapy has been extensively studied [13]. Pre-treatment with a high dose of ranitidine has been shown to reduce both AUC and Cmax of gefitinib [20]. However, other studies reported no significant interactions between ARAs and gefitinib [21,22].

Afatinib is an irreversible blocker of the ErbB (EGFR) family, inhibiting the activity of EGFR-1, -2 (HER2), and -4, as well as the transphosphorylation of ErbB3 [12]. Because afatinib is highly soluble from pH 1 to 7.5, no interactions with ARAs are expected [23]. On the contrary, dacomitinib, another irreversible EGFR-TKI, has pH-dependent solubility and concomitant administration with ARAs should be avoided [13].

Osimertinib is a third-generation orally available, potent, EGFR-TKI that selectively inhibits the activity of mutant forms of EGFR, including T790M [24,25]. When healthy volunteers were given 80 mg of osimertinib, no impact on its serum concentrations were observed among those also receiving omeprazole [26].

### 2.2. Anti-VEGF Inhibitors

Several vascular endothelial growth factor receptor (VEGF) TKIs are approved for oncologic use, including axitinib, cabozantinib, lenvatinib, pazopanib, regorafenib, sorafenib, sunitinib, and vandetanib. Most oral VEGF-TKIs undergo hepatic metabolism by cytochrome P450 3A4 [27]. Although not yet proved, gastric pH may interfere with systemic absorption of and potential exposure to certain VEGF-TKIs [28].

Sunitinib is an orally administered TKI which potently inhibits VEGFR, platelet-derived growth factor receptor (PDGFR), and c-Kit. Its optimal solubility depends on a gastric pH ranging from 1.2 to 6.8, above which solubility drops exponentially, leading to reduced absorption [9]. In a retrospective analysis including 847 kidney cancer patients, no significant association between the risk of death and concomitant TKI-PPI use was observed [17]. Another pooled analysis of patients with metastatic renal cell cancer who participated in several clinical trials showed no interaction between PPI users and non-users. In this study, 952 patients were treated with sunitinib, 624 with axitinib and 160 with sorafenib, and, in total, 120 individuals were PPI users at baseline (5.5%). For the entire cohort, no differences in both OS and PFS were observed between PPI users and nonusers [28]. Nonetheless, a more recent retrospective review with 383 patients came to a different conclusion, in which OS was decreased among patients receiving concomitant sunitinib and ARAs compared with non-users [29].

Pazopanib also inhibits VEGFR-1, -2, and -3, PDGFR-α and -β, and KIT [30]. Its absorption is pH-dependent, and the drug is almost insoluble at pH > 4 [9]. In a DDI study, the combined use of pazopanib and esomeprazol led to a decrease in the pazopanib Cmax and AUC [31]. In a pooled analysis of patients with advanced soft tissue sarcoma who were prescribed pazopanib in the European Organisation for Research and Treatment of Cancer (EORTC), 62,043 and 62,072 revealed worse PFS and OS among ARA users versus nonusers [30]. On the other hand, a retrospective cohort study of 90 patients with metastatic renal cell carcinoma treated with pazopanib showed no difference with respect to PFS or OS among ARA users versus non-users [32].

Axitinib is a small molecule indazole derivative, which is an oral, potent multi-target TKI of VEGFR-1, -2 and -3. In a phase I trial, the use of the potent PPI rabeprazole decreased axitinib absorption (reduction in Cmax), but the extent of axitinib absorption was unaffected (marginal to negligible changes in the AUC) [33].

Cabozantinib, an inhibitor of c-MET, VEGFR, AXL and other kinases, exhibits a pH-dependent solubility profile in vitro [34]. A phase I clinical pharmacology study aimed to observe DDIs between cabozantinib and esomeprazole among healthy subjects. AUCs and Cmax were similar with or without the PPI, implying that concomitant use of PPIs or weaker ARAs with cabozantinib is probably safe [35].

Lenvatinib is a multi-kinase inhibitor of VEGFR-1, -2 and -3, fibroblast growth factor receptor family (FGFR1-4), PDGFR-alpha, KIT, and rearranged during transfection receptor (RET) [36]. According to the American and European regulatory agencies, FDA and EMA respectively, there are no studies evaluating concomitant use of lenvatinib with ARAs, but it has been considered safe due to a physiologically based pharmacokinetic model [37]. Sorafenib, a multi-protein kinase inhibitor with activity against VEGFR, PDGFR and RAF kinases, is also safely co-administered with ARAs [37].

Regorafenib is also an oral multi-kinase inhibitor targeting angiogenic, stromal, and oncogenic tyrosine kinase receptors (VEGFR, KIT, B-Raf, PDGFR, and FGRF) [38]. Recently, a randomized crossover trial demonstrated that regorafenib and esomeprazole may be safely combined in clinical practice, irrespective of the time of esomeprazole administration [39].

Vandetanib, a selective inhibitor of VEGFR, EGFR and RET signaling, is currently indicated for the treatment of medullary thyroid cancer. A phase I study conducted among healthy volunteers confirmed that exposure to vandetanib was unchanged during coadministration with either omeprazole or ranitidine [40].

### 2.3. mTOR Inhibitors

mTOR inhibitors are a class of drugs that inhibit the mammalian target of rapamycin (mTOR), including everolimus and temsirolimus. Despite the present scarce data on the influence of PPIs on mTOR inhibitors, no identified interactions of risk level A or greater has been found between those classes of drugs [27]. The metabolizing process of PPIs by cytochrome P450 (CYP) leads to a competitive inhibition of these enzymes which might affect the metabolism of mTOR inhibitors, which are also metabolized by CYP3A4 [10,41].

In a study conducted among liver transplant recipients who received mTOR inhibitors as immunosuppressive agents and were on oral therapy with pantoprazole 40 mg OD, pantoprazole (which has a lower binding capacity to CYP2C19] did not significantly affect the serum trough levels of tacrolimus, everolimus or sirolimus [42].

### 2.4. BRAF Inhibitors/MEK Inhibitors

Vemurafenib and encorafenib, which are inhibitors of oncogenic BRAF kinase [43], are commonly used in melanoma with BRAF mutation. The prevalence of PPI use ranged from 14.2% to 29.2% in melanoma patients in two primary data sources of the two largest healthcare systems in the US [7]. Interaction between PPIs and vemurafenib leads to a risk of its underexposure due to changes in gastric pH [7,44].

With the aim of investigating the potential DDI between ARAs and vemurafenib in BRAF-mutant melanoma patients, a large retrospective analysis from 4 phase III trials was conducted. Of 920 patients, 31.8% reported concomitant use of an ARA (≥85% PPI). There were no significant differences in terms of treatment efficacy according to ARA use or not. Furthermore, for those patients with available vemurafenib concentration data, its concentration was similar regardless of ARA use [44]. Another retrospective study with 112 melanoma patients evaluated the clinical impact of concomitant administration of ARAs and vemurafenib. A trend towards worse PFS was observed among those who used both drugs [45]. Until more definitive data becomes available, concomitant use of PPIs and BRAF inhibitors should be discouraged.

Cobimetinib, binimetinib and trametinib are all potent and selective inhibitors of MEK1/2. Studies conducted in healthy subjects revealed delayed absorption of cobimetinib with concomitant rabeprazole use. However, no significant effects on Cmax and AUC in the fasted state were noted [46]. Until further studies are available, cobimetinib may be safely used with ARAs.

A phase I study is currently ongoing evaluating DDI of encorafenib and binimetinib on probe drugs, including omeprazole, in patients with advanced BRAF mutated solid tumors [47].

### 2.5. MET Inhibitors

Cabozantinib is an orally administered TKI with activity against VEGFR2, FLT3, c-KIT, RET and MET [48]. It is characterized by low solubility, high permeability, and exhibits a pH dependent solubility profile in vitro (practically insoluble at pH > 4) [48].

The effect of esomeprazole on the PK of cabozantinib was evaluated in a study with 22 patients. The mean plasma peak concentration and overall exposure was similar regardless of the PPI use. Despite the trial’s small sample size, it indicates that cabozantinib and PPIs may be used concomitantly without compromising the efficacy [35].

Crizotinib is also a multi-kinase inhibitor, including activity against c-Met and ALK. The solubility in aqueous media decreases over the range of pH 1.6 to pH 8.2 from greater than 10 mg/mL to less than 0.1 mg/mL [49]. Because the solubility of crizotinib is pH dependent, decreasing at higher pH, the impact of PPIs on crizotinib absorption cannot be fully excluded. Thus, future studies are necessary to investigate the interaction between crizotinib and PPIs.

### 2.6. ALK Inhibitors

Crizotinib (already discussed), alectinib and brigatinib are orally bioavailable TKIs that inhibit anaplastic lymphoma kinase (ALK). Alectinib also inhibits RET, while crizotinib inhibits MET [50]. Brigatinib has selective activity against ALK and ROS1 [50]. Besides, it also has activity against *EGFR* mutations, including the T790M mutation [50].

In a study with healthy subjects, coadministration of esomeprazole and alectinib had no relevant effect on the exposure of the latter [51]. No studies are currently available regarding concomitant use of brigatinib and PPIs.

### 2.7. TRK Inhibitors

Larotrectinib is an inhibitor of tropomyosin receptor tyrosine kinases (TRK), including TRKA, TRKB, and TRKC while entrectinib has activity against ALK, ROS1, and TRKA, TRKB, and TRKC proteins [52]. There are no studies so far investigating DDIs between TRK inhibitors and PPIs.

### 2.8. HER2 Inhibitors

Lapatinib is a potent and reversible TKI of both EGFR type 2 (HER2/ERBB2] and EGFR type 1 (HER1/ERBB1] [53]. The concomitant use of lapatinib and PPIs may alter the serum concentration of lapatinib due to changes in gastric pH [53]. Since the aqueous solubility of lapatinib declines significantly at pH > 4, its bioavailability might be reduced by ARAs [54]. In study subjects, coadministration with the PPI esomeprazole (40 mg once daily for 7 days) decreased lapatinib exposure by an average of 27% (range 6% to 49%). Nonetheless, the clinical significance of this effect remains unknown and no labeled warning has been generated [53,55]. Whenever possible, long-term suppression of gastric acid secretion should be avoided.

### 2.9. Phosphatidylinositol 3-Kinase Inhibitors

Alpelisib is an orally available phosphatidylinositol 3-kinase (PI3K) inhibitor which acts against PI3K-alpha. It was recently approved in several countries for the treatment of hormone-positive metastatic breast cancer harboring a PI3K mutation [27,56]. According to the label, alpelisib can be co-administered with ARAs. Concomitant use of ranitidine with 300 mg of alpelisib led to its reduced absorption and overall exposure [56]. However, to this moment, no description of interaction between alpelisib and PPIs is found in the literature [27,56].

### 2.10. KIT Inhibitors

Imatinib mesylate is an orally active TKI that inhibits the Bcr-Abl tyrosine kinase, c-KIT and PDGFR [57]. Common adverse events associated with imatinib administration include dyspepsia and nausea, leading PPIs to be frequently co-administered. It has been known that imatinib is a substrate for ATP-binding-cassette transporters, which are antagonized by PPIs. Therefore, imatinib absorption may be compromised with PPI concurrent administration [58]. In a similar fashion, dasatinib, also a Bcr-Abl TKI, is only half absorbed when in conjunction with ARAs [59].

In a study with 12 healthy subjects, the pharmacokinetics of imatinib with concomitant administration of omeprazole was not altered [60]. According to this study, omeprazole may be safely co-administered with imatinib without affecting the absorption of imatinib [60]. However, those findings should not be extrapolated to other PPIs. Preclinical studies with pantoprazole revealed altered clearance mechanisms of imatinib [61]. In addition, dermatologic adverse effect of imatinib may be enhanced in the presence of lansoprazole [62], although the real mechanism for interaction remains unknown.

## 3. Monoclonal Antibodies

### 3.1. Anti-EGFR Monoclonal Antibodies

Cetuximab and panitumumab are monoclonal antibodies that bind to the human EGFR [63,64]. A retrospective study with 118 patients explored the influence of PPIs on cetuximab skin toxicity and also the synergistic effect of hypomagnesemia of these drugs [65]. Skin toxicity of any grade was more frequent in patients receiving concomitant PPI (56.9 vs. 36.7%, *p* = 0.08). Grade 3–4 skin toxicity was reported in 32.8% patients on PPIs compared to only 3.3% of patients not on PPIs (*p* = 0.001, HR = 11.88, 95%CI = 2.76–51.07). In addition, hypomagnesemia was reported in 25.9% of patients receiving concomitant PPIs compared with 10.4% of patients not on PPIs (*p* = 0.08) [65].

Hypomagnesemia is a frequent adverse reaction with anti-EGFR monoclonal antibodies and is related to treatment duration [66]. Concomitant administration of PPIs and anti-EGFR monoclonal antibodies may influence the absorption of oral magnesium since gastric pH is a crucial factor. PPIs are known to significantly reduce serum magnesium level and the FDA warns of an association between hypomagnesemia and PPIs [67].

For this reason, coadministration of PPIs and EGFR inhibitor therapy may lead to DDIs, delayed elimination and, potentially, increased toxicity if not monitored appropriately. Further investigations are necessary to understand the mechanism of increased cetuximab skin toxicity with the concomitant use of PPI. Furthermore, prospective studies are necessary to confirm the possible interaction between anti-EGFR therapy and PPIs.

### 3.2. Anti-Angiogenic Monoclonal Antibodies

Bevacizumab is a recombinant humanized monoclonal antibody that selectively binds to circulating VEGF while ramucirumab is a human IgG1 monoclonal antibody which selectively binds to the extracellular VEGFR-2 [68,69]. Aflibercept is a recombinant fusion protein which is comprised of portions of binding domains for VEGFR 1 and 2, attached to the Fc portion of human IgG1 [70].

Until now, no DDIs have been found between anti-angiogenic monoclonal antibodies and PPIs in the literature. This does not necessarily mean no interactions exist and studies are urgently needed to better investigate whether PPIs can negatively affect these drugs’ efficacy.

### 3.3. Anti-HER2 Monoclonal Antibodies

Trastuzumab is a monoclonal antibody which binds to the extracellular domain of HER2 while pertuzumab is a recombinant humanized monoclonal antibody that blocks a different antigenic region of the HER2 extracellular dimerization site with other HER family members [71,72]. There are no known DDIs between anti-HER2 monoclonal antibodies and PPIs in the literature.

## 4. Anti-Hormonal Agents

### 4.1. Estrogen Receptor Inhibitors

Tamoxifen and fulvestrant are competitive inhibitors of estrogen binding to estrogen receptors (ERs). No interaction between PPIs and either tamoxifen or fulvestrant has been described so far. Aromatase inhibitors act by blocking the last step in biosynthesis of estrogens, which is the conversion of androgens to estrogens by the heme-containing enzyme, aromatase. There are no studies correlating the use of PPI with negative impact on efficacy of aromatase inhibitors, such as anastrozole, letrozole, and exemestane.

Nonetheless, there is a significant relationship between chronic use of PPI and bone mineral density [BMD) loss [73,74,75]. A prospective study with over 1200 post-menopausal women showed that the use of PPIs was an independent risk factor for vertebral fractures. This risk can be increased in patients using aromatase inhibitors for breast cancer, since these medications are also known to accelerate BMD losses and osteoporosis at a rate up to 2.6% per year of bone loss [76]. An American cohort study with 9138 patients showed that around 10% of breast cancer patients were using a PPI at the time of starting an aromatase inhibitor [77]. In a similar fashion, patients receiving gonadotropin releasing hormone (GnRH) agonists, such as goserelin, are at an increased risk of developing osteoporosis with concomitant PPI use.

### 4.2. Androgen Receptor Inhibitors

Enzalutamide is an androgen receptor signaling inhibitor. Pharmacological studies have found that enzalutamide can decrease the plasma levels of PPIs metabolized through CYP2C19 (such as omeprazole) by close to 70% [78]. It has been postulated that co-medication of enzalutamide and PPIs may lead to faster metabolism of the latter, resulting in lower plasma levels and potential suboptimal efficacy.

Abiraterone is an inhibitor of 17α-hydroxylase/C_17,20_-lyase (CYP17), an enzyme necessary for androgen synthesis. Up to now, no interactions are known between abiraterone and PPIs.

## 5. Immunotherapy

The richness of human gut microbiome composition [79] and its effect on immune system has been explored in the last years. Preclinical studies described gut microbiome affecting tumor responses to both chemotherapy and immunotherapy [80]. The field of microbiome research highlights the importance of microbiota integrity to the host immunomodulatory response [81]. Thus, there is an unarguable and complex linkage between microbiota, immune system, and responsiveness to cancer treatments.

It is recognized that PPI use may affect and change gut microbiome [11,82], due to their association with enteric infections, such as Clostridium difficile [83,84] and other pathogens’ colonization. Similar alterations are observed with antibiotics use. This disequilibrium of commensal microbiota is related to underlying decrease of diversity and enable a status of chronic inflammation and tumor microenvironment [85]. Checkpoint inhibitors are one of the major breakthroughs in the recent years in cancer treatment and being affected by intestinal gut disruption is remarkably startling.

In a recent study, the gut microbiome of melanoma patients seems to modulate anti-PD1 response [86]. The authors evaluated oral and gut microbiome samples from 112 patients treated with immunotherapy. Responders presented significant differences in the composition and enriched microbiome diversity compared to non-responders, suggesting a link between favorable gut flora profile and enhanced anti-tumor response. An abundance of Ruminococcaceae bacteria in the feces of responders was identified [86].

In a retrospective analysis from CheckMate 069 phase II trial, 140 patients with advanced melanoma on immunotherapy (either ipilimumab alone or in combination with nivolumab) were evaluated regarding PPI use and outcomes [87]. The objective response measured in patients receiving immunotherapy and PPIs was approximately half of those patients were not on PPIs. PFS and OS were also reduced among PPI users. The negative effect of PPIs was also confirmed in an independent cohort of patients treated with anti-PD1 (pembrolizumab or nivolumab) monotherapy. Based on these data, avoidance of PPIs concomitant to immunotherapy was recommended [87].

In addition, an exploratory analysis from POPLAR and OAK trials, including patients with advanced NSCLC randomized to atezolizumab or docetaxel as second line treatment, revealed poorer outcomes in individuals who were receiving PPIs. A significantly higher risk of death was observed among PPI users (HR = 1.26). In the atezolizumab arm, patients on PPIs had a median OS and PFS of 9.6 and 1.9 months compared to 14.5 and 2.8 months in non-users, respectively [88].

However, not all studies confirmed negative impact of PPI use concomitant with immunotherapy. A study examined whether gut dysbiosis associated with the presence of antibiotics or PPIs could affect anti-PD1 or anti-CTLA4 efficacy. In mice models, coadministration of antibiotics and immunotherapy led to increased tumor size and decreased survival. In contrast to antibiotics use, concomitant PPI use had no detrimental effect on PFS and OS [89].

A retrospective single center study with 158 patients attempted to investigate whether PPIs (used by 46.2% of individuals) could modulate the efficacy of anti-PD-1/PD-L1 therapies in cancer patients [88]. Despite several limitations, there were no statistically significant differences between PPI users and non-users in terms of PFS and OS [90]. So it remains unclear whether concomitant use of PPIs might interact negatively with immunotherapy.

## 6. Cycline Inhibitors

Cyclin-dependent kinases (CDK) are important regulatory enzymes controlling cell cycle and cell division. CDK 4/6 inhibitors, such as palbociclib, ribociclib, and abemaciclib, have been successfully used in many malignancies, especially breast cancer. For ribociclib and abemaciclib, no interactions have been described with ARAs [27,91]. Given the fact that ribociclib has its highest solubility below pH 4.5, no DDIs are expected with PPIs [91,92].

On the other hand, the solubility of palbociclib is reduced at pH values above 4. A PK study reported that co-administration of rabeprazole and palbociclib under fasting conditions decreased both Cmax and AUC [93]. Palbociclib therefore should be taken with food and the impact of PPI interaction is minimal [94]. Given the current evidence, CDK inhibitors may be safely used with PPIs.

## 7. PARP Inhibitors

Olaparib, talazoparib, rucaparib, niraparib and veliparib are inhibitors of the enzyme poly ADP ribose polymerase (PARP). Binding to PARP inhibits single stranded DNA base excision repair and creates PARP-DNA complexes that lead to double-stranded DNA breaks, ultimately causing cell death in tumors that cannot repair double stranded breaks reliably, such as those with BRCA 1 or 2 mutations [95]. There are no known DDIs between PARP inhibitors and PPIs in the literature.

## 8. Oral Chemotherapeutic Agents

Capecitabine is an oral pro-drug of 5-fluorouracil [96]. Approximately 1.5 h after ingestion, it reaches peak plasma concentration, dissolving quickly and being absorbed predominantly in the upper gastrointestinal tract [96]. It has been postulated that increase in the gastric pH may lead to reduced dissolution and absorption of capecitabine tablet, although in vitro data has not supported this so far [97]. Although pK studies have been conducted on the effect of PPIs on capecitabine, concurrent anti-acid Maalox use has indeed reported small increases in systemic capecitabine levels [98].

The only clinical data on PPI-capecitabine interaction is based on two retrospective post hoc analyses of randomized controlled studies. In the randomized phase III TRIO-013/LOGIC trial, which compared capecitabine and oxaliplatin (CAPOX) with or without lapatinib in HER2–positive metastatic gastroesophageal cancer patients, lower PFS (4.2 vs. 5.7 months, *p* < 0.001) and OS (9.2 vs. 11.3 months, *p* < 0.04] was identified in the group of patients who received concomitant PPI. However, the study did not collect plasma drug levels from patients, making it unfeasible to draw any direct pK explanation [99].

In a study with colorectal cancer patients treated with adjuvant capecitabine, PPI users had a decreased 5-year recurrence free survival (74 vs. 83%). Nonetheless, after multivariate analysis, this difference became insignificant [100]. Given the current uncertainties related to the concomitant use of PPIs and capecitabine, until further data is available, deprescribing PPIs should be considered whenever capecitabine is recommended. When an ARA is necessary, Maalox should be preferred.

Temozolamide is an oral prodrug, delivering a methyl group to purine bases of DNA [100], while lomustine is an orally administered nitrosurea [101] and cyclophosphamide is an oral type of nitrogen mustard. [102] These are all alkylating agents. There is no described interaction between these drugs and PPIs in the present literature.

Vinorelbine is a semisynthetic vinca alkaloid, which inhibits cell growth by binding to the tubulin of the mitotic microtubules. [103] while etoposide is a DNA topoisomerase II inhibitor [104]. There is also no described interaction between PPIs and those drugs in the literature.

## 9. Parenteral Chemotherapeutic Agents

For all parenteral cytotoxic chemotherapy, except methotrexate, no interactions were found with PPIs. Methotrexate (MTX) is an antifolate drug that inhibits dihydrofolate reductase [105].

Several published case reports describe patients who experienced altered methotrexate pharmacokinetics (i.e., increased concentrations and/or delayed elimination) and/or excessive toxicities associated with concurrent PPI, such as omeprazole, esomeprazole, or pantoprazole [106,107,108,109,110,111,112]. In most of these cases, the abnormal methotrexate concentrations/elimination were not evident with prior or subsequent administration of methotrexate without the concurrent PPI [106,107,108,109,110].

Finally, a retrospective analysis of the impact of PPIs on 73 cycles of high dose methotrexate in 43 individuals showed that patients who received a PPI with methotrexate had significantly higher methotrexate levels at 48 and 72 h [113]. The mechanism for this possible interaction is uncertain, but some have been proposed. Methotrexate is actively secreted in the distal renal tubules with hydrogen ions produced via the hydrogen/potassium ATPase pump. PPIs, by inhibiting renal elimination of the hydrogen ion, may inhibit methotrexate elimination. Data from in vitro and animal studies support at least some role for PPI-mediated inhibition of the breast cancer resistance protein (BCRP, ABCG2] and/or MRP2 transporters [114,115].

## 10. Conclusions

The effect of PPIs on the efficacy and safety of anticancer agents requires extensive investigation. There is a caveat, and randomized controlled trials should rapidly evolve to elucidate this issue of every oncologist’s daily practice. Meanwhile, we encourage healthcare providers to limit unnecessary overuse of PPIs. 

## Figures and Tables

**Table 1:** Interactions between Oncologic Drugs and Proton Pump Inhibitors.

Oncologic Treatment Drugs		Type of Evidence on Interactions			
Class	Name	Decreased Activity and/or Increased Toxicity	No Interaction	Controverse Data	Absence of Data
Epidermal growth factor receptor (EGFR) tyrosine-kinase inhibitors (TKIs)	Erlotinib	✓			
	Gefitinib			✓	
	Afatinib				✓
	Dacomitinib				✓
	Osimertinib		✓		
Anti-vascular endothelial growth factor (VEGF) inhibitor	Sunitinib		✓		
	Pazopanib			✓	
	Axitinib		✓		
	Cabozantinib		✓		
	Levantinib				✓
	Sorafenib				✓
	Regorafenib		✓		
	Vandetanib		✓		
Mammalian target of rapamycin (mTOR) inhibitor	Everolimus		✓		
	Sirolimus		✓		
	Tacrolimus		✓		
	Temsirolimus				✓
BRAF/MEK inhibitor	Vemurafenib			✓	
	Cobimetinib		✓		
	Encorafenib				✓
	Trametinib				✓
	Binimetinib				✓
MET/anaplastic lymphoma kinase (ALK) inhibitor	Crizotinib				✓
ALK inhibitor	Alectinib		✓		
	Brigatininib				✓
Tyrosine kinase (TRK) inhibitor	Larotrectinib				✓
HER2 inhibitor	Lapatinib			✓	
PI3K inhibitor	Alpelisib				✓
KIT inhibitor	Imatinib	✓ (a)			
Anti-EGFR mAbs	Cetuximab	✓ (b)			
	Panitunumab	✓ (b)			
Anti-angiogenic mAb	Bevacizumab				✓
	Ramucirumab				✓
Anti-HER2 mAb	Trasntuzumab				✓
	Pertuzumab				✓
Estrogen receptor inhibitor	Tamoxifen	✓ (c)			
	Fulvestrant	✓ (c)			
	Anastrozole	✓ (c)			
	Letrozole	✓ (c)			
	Exemestane	✓ (c)			
Androgen receptor inhibitor	Enzalutamide	✓ (d)			
	Abiraterone				✓
Immunotherapy	Pembrolizumab			✓	
	Nivolumab			✓	
	Ipililimab			✓	
	Atezolizumab			✓	
Cycline inhibitor	Ribociclib		✓		
	Abemaciclib				✓
	Palbociclib				✓
Poly ADP ribose polymerase (PARP) inhibitor	Olaparib				✓
	Talazoparib				✓
Oral QT	Capecitabine			✓	
	Temozolamide				✓
	Lomustine				✓
	Cyclophosphamide				✓
	Vinorelbine				✓
	Etoposide				✓
IV QT	Methotrexate	✓			
	Other IV QTs				✓

(a) Enhanced dermatologic adverse effects (only for lanzoprazol), (b) increased skin toxicity and hypomagnesemia, (c) accelerated bone mineral density (BMD) loss, (d) decreased plasma level of PPIs. Mechanisms of drug–drug interactions DDI for each class of drugs are elucidated in the main text.
